# Behavioral Measures in a Cognitive-Motor Batting Task Explain Real Game Performance of Top Athletes

**DOI:** 10.3389/fspor.2020.00055

**Published:** 2020-05-12

**Authors:** Daiki Nasu, Masumi Yamaguchi, Akemi Kobayashi, Naoki Saijo, Makio Kashino, Toshitaka Kimura

**Affiliations:** NTT Communication Science Laboratories, Nippon Telegraph and Telephone Corporation, Kanagawa, Japan

**Keywords:** movement onset time, motion capture, hitting motion, path-analysis, button pressing

## Abstract

Excellent athletic performance in baseball and softball batting is achieved through the momentary cognitive-motor processes. However, in previous studies, cognitive and motor processes are investigated separately. In this study, we focused on the difference in the time of swing onset (a delta onset) during a batting task where 17 elite female softball batters hit balls randomly thrown at two different speeds by pitchers. The delta onset included both cognitive and motor processes because the batters needed to anticipate the ball speed and discriminate their swing motion according to the time-to-contact. Then, we investigated the relationship between the delta onset and the batting outcomes of the batting task, and the relationship between the experimental outcomes and actual batting performance (batting average) over a season. We used path analysis to clarify the structure of the cognitive-motor processes and consequent performance. We found that the batters who had a larger delta onset attained superior batting outcomes (i.e., higher exit velocity and lower miss ratio) in the batting task, and these experimental outcomes explained 67% of the batting average in real games. On the other hand, the cognitive scores (judgement accuracy and rapidity) obtained from a button pressing task, where batters responded to a ball by pressing a button instead of actually swinging, explained only 34% of the batting average. Therefore, our model quantitatively describes the key cognitive-motor structure for athletes and can partially predict a batter's performance in real games. These findings suggest that it is important to employ both cognitive and motor processes in performing tasks, such as this batting task, to properly evaluate a batter's actual ability.

## Introduction

Human action is strongly related to cognitive processes such as sensory processing, prediction, and decision making. These processes closely affect and restrain motor behavior associated with motor planning and execution (Wolpert and Flanagan, 2001). In sports, such cognitive-motor processes frequently occur in a split second. For example, when batting in baseball, softball, and cricket, it takes around 500 ms or less for the ball to cover the distance between the pitcher and batter. In this amount of time, a batter must estimate precisely when and where the ball is going to cross the plate and decide whether or not to swing (a cognitive process) and then synchronize the swing to make contact with the ball (a motor process).

Many studies in sports biomechanics and training science have investigated multiple aspects of batting motion, such as kinematics (Welch et al., 1995; Escamilla et al., 2009; Inkster et al., 2011; Iino et al., 2014) and kinetics (Shaffer et al., 1993; Nakata et al., 2013). These studies, where participants swung against a stationary ball or a ball traveling at a constant speed, characterized the physiological and mechanical factors required for powerful and accurate action but did not address the cognitive aspects. On the other hand, sports psychology and neuroscience studies have described cognitive processes in athletes, such as the ability to anticipate ball trajectory and/or the opponent's kinematics (Abernethy, 1990; Paull and Glencross, 1997; Renshaw and Fairweather, 2000), and the parts of the brain activation in the perceptual decision-making processes (Radlo et al., 2001; Muraskin et al., 2015). While these studies focused on experts' cognitive functions, they examined them in simple and unrealistic environments, where participants reacted by pressing a button while watching a video of a pitcher's motion or a visual stimulus. Although these studies showed the importance of both cognitive and motor processes, these were investigated separately.

Some studies investigated the swing motion of baseball batters attempting to hit an approaching ball in simulation (Gray, 2002, 2009, 2010; Ranganathan and Carlton, 2007; Gray and Cañal-Bruland, 2018). These studies focused on the temporal accuracy of the swinging motion in varying pitch speeds in order to evaluate the batters' ability to anticipate. The different ball speeds resulted in different ball flying times (time to contact: TTC); therefore, the batters were required to predict the TTC and initiate and adjust their swing accordingly. In particular, the swing onset is considered to reflect the first decision of the batter with regards to motor execution. That is, the difference in the time of swing onset when hitting a ball thrown at different ball speeds would be one of the key factors reflecting the cognitive-motor processes. However, it has also been reported that there are two types of strategies (onset control vs. ongoing adjustment) used in response to different TTCs in a batting-simulated rapid interceptive task (Ijiri et al., 2014). This suggests the possibility that batters could achieve high performance by adjusting their motion in an ongoing fashion even if they cannot delay their swing onset according to TTCs. Therefore, it is necessary to investigate the direct relationship between the swing onset and the batting outcome. However, this relationship is not yet clear because previous studies were conducted through simulated batting (Gray, 2002, 2009, 2010; Ranganathan and Carlton, 2007; Gray and Cañal-Bruland, 2018) or by only elbow flexion (Ijiri et al., 2014).

Moreover, most studies have dealt only with data obtained in the laboratory and these did not examine the relationship of experimental results with the actual performance in real games. Determining the extent to which results obtained in the laboratory can explain actual performance would be extremely valuable and highly interesting to athletes and coaches.

With this, the current study aimed to clarify the relationship between the difference in the time of swing onset (a delta onset) and the batting outcomes within a real batting experiment, where softball batters attempted to hit balls thrown at two speeds (fastball and slowball) by real pitchers. We also investigated the relationship between these experimental batting outcomes and actual batting performance obtained from real games (batting average). To clarify the structure of cognitive-motor processes and consequent performance, we performed path analysis using structural equation modeling (SEM). Based on the model, it was expected that the delta onset would influence experimental batting outcomes, and that these experimental outcomes would explain individual batting average in real games.

As was mentioned earlier, psychology and neuroscience studies have conventionally used basic tasks such as a button pressing task to evaluate cognitive function. However, it is unclear to what extent the scores of these basic tasks explain the actual performance in real games. There is skepticism regarding the extent to which the results from unrealistic measurements can be applied to actual complex situations such as in sports (Ranganathan and Carlton, 2007; Güldenpenning et al., 2017). Therefore, we further conducted another experiment (Go/Nogo button pressing task) to investigate the relationship between cognitive scores from this experiment and the batting average in real games. This allows us to examine to what extent cognitive scores derived from this test would explain the batting average compared to the outcome of the batting task.

While batting in softball is similar to that of baseball and cricket, the time constraints are more severe in softball because there is less distance between the pitcher and the batter. Therefore, the temporal adjustment to the ball investigated in this study is relatively meaningful.

## Materials and Methods

### Participants

Seventeen elite female softball batters participated in the experiments. They are competitive fast pitch softball players who belong to the Japan softball top league, and four of them were members of the Japan national team. The mean ± SD age was 23.2 ± 4.0 years and the mean number of years that they have played softball and/or baseball was 15.9 ± 4.9 years. Seven of them were right-handed batters and all of them were fielders; none were pitchers. The mean ± SD height was 163.7 ± 4.9 cm. All the participants provided written informed consent prior to the experiments. This study was approved by the Ethics and Safety Committees of NTT Communication Science Laboratories and were in accordance with the Declaration of Helsinki.

Two female softball pitchers (pitchers A and B) from the same team as the batters volunteered to act as pitchers in the experiments. Both were right-handed pitchers. Each batter faced one of the two pitchers. Nine batters faced pitcher A and eight batters faced pitcher B. The pitch velocity of a fastball immediately before bat-ball contact did not differ between the pitchers (mean ± SD: 82.0 ± 5.4 km/h for A; 83.0 ± 0.9 km/h for B; two sample *t*-test, *p* = 0.61) nor did that of a slowball (60.0 ± 3.4 km/h for A; 59.4 ± 1.7 km/h for B; two sample *t*-test, *p* = 0.66). Furthermore, the batters' responses to pitchers A and B did not differ in the delta onset (23.5 ± 6.5 % for A; 26.9 ± 6.8 % for B; two sample *t*-test, *p* = 0.31), in the exit velocity (96.7 ± 6.5 km/h for A; 98.0 ± 7.5 km/h for B; two sample *t*-test, *p* = 0.71), and in the miss ratio (0.14 ± 0.21 for A; 0.10 ± 0.14 km/h for B; two sample t-test, *p* = 0.61) (see Data Analysis for the definitions of each variable). Thus, our study was based on the premise that the difficulty to hit the pitches did not differ depending on the pitcher.

### Batting Task

#### Task and Apparatus

The experiments were conducted on an indoor field at our laboratory. After a sufficient warm-up period and several practice hits, the batters were asked to hit fastballs and slowballs randomly thrown by the pitchers. Each batter swung 10 times for each pitch type, and the pitch type was not announced beforehand. Batters were instructed to swing only when the pitched balls were in the strike zone. Foul balls were excluded from the analysis. In total, 14.9 ± 3.0 swings were analyzed per batter. The batter's motion was measured using nine axes of inertial sensors attached to various parts of the body (MVN BIOTECH, Xsens B.V.) with a sampling frequency of 240 Hz. Ball movement around the time of bat-ball contact (ball impact) was recorded using two high-speed cameras with a sampling frequency of 240 Hz (Sports Coaching Cam, JVCKENWOOD Corp.). These cameras were placed diagonally at 45° in front of the batter, 10 m away from the home plate and protected with screens. The pitcher's camera was also used to detect the pitcher's ball release. The system was synchronized by a start signal from the motion capture system, which lit LEDs installed in front of the three cameras.

#### Data Analysis

##### Delta onset

**Figure 1:**
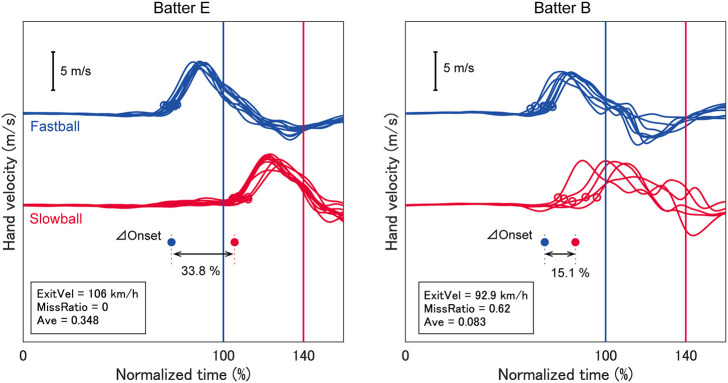
Temporal patterns of hand velocity. The hand velocities in the pitcher's direction for two typical batters are shown. The time between ball release and mean impact was normalized as 100% for a fastball and 140% for a slowball. Open circles indicate swing onsets and filled circles represent individual mean values. The exit velocity, miss ratio and batting average for each batter are indicated in each panel. One batter exhibited a large delta onset and higher performance (batter E), but the other batter exhibited a small delta onset and lower performance (batter B).

In this study, the TTC was normalized to eliminate inter- and intra-personal variations. TTC was defined as the elapsed time between the ball being released by the pitcher and its crossing the mean impact position for each batter. Because each batter stood differently in the batter's box, we used the mean impact position for each batter. The TTCs across all analyzed trials were 444.9 ± 17.7 ms for fastballs and 621.6 ± 26.6 ms for slowballs. The TTC for a fastball and slowball was normalized as 100 and 140%, respectively, because the pitch velocities and TTCs of fastballs of both pitchers were ~1.4 times faster than those of slowballs (Figure 1).

We analyzed the batter's swing onset time based on the temporal patterns of hand velocity. Hand velocity was defined as the velocity of the hand segment on the catcher's side (i.e., the right hand for a right-handed batter) relative to the velocity of the pelvis segment in the pitcher-catcher direction. This was done to remove the translation component of the trunk from the swing onset. Swing onset was defined as the moment at which the hand velocity exceeded a certain threshold, which was 10% of the mean peak velocity for each batter (0.61 ± 0.09 m/s) (open circles in Figure 1). The threshold was determined so that early phase fluctuations were not erroneously defined as the onset. The delta onset for each batter was calculated as the difference between the mean times of the swing onset for fastballs and slowballs (filled circles in Figure 1), presented as normalized time (%).

##### Exit velocity

The ball trajectory around the time of bat-ball impact was obtained by manually digitizing video images using the MATLAB code (Hedrick, 2008). Three-dimensional coordinates were calculated using the direct linear transformation method with a 2 × 2.5 × 1.5 m calibration frame with 90 reference points at 0.5-m intervals. The pitch velocity and the exit (batted) velocity for each trial were calculated as the mean velocity of five frames (20.8 ms) immediately before and after ball impact. The exit velocity that flew into the infield was averaged for each batter (km/h).

##### Miss ratio

The miss ratio for each batter was calculated as the number of missed swings divided by the total number of swings for both pitch speeds. A missed swing means that the batter swung but could not hit the ball.

##### Swing velocity at stationary ball

This variable was used in an additional analysis in the results section (see section Comparison of Batting Outcomes Between Groups With Large and Small Delta Onsets). The swing velocity at stationary ball was measured using an easy-to-use instrument designed for swing analysis (Swing Tracer, MIZUNO Corp.). Batters hit three stationary balls on a tee stand. The bat velocities immediately before bat-ball impact were averaged for each batter (km/h).

##### Batting average

The batting average is the most general index for evaluating a batter's ability in a real game performance, and values were taken from the same season as when the experiments were conducted (2017 season). This variable was calculated as the number of hits divided by the times at bat obtained from the results of official and practice matches against teams in the same league (30 matches in total).

### Button Pressing Task

#### Task and Apparatus

In the button pressing task, the batters faced the same pitcher as in the batting task in a separate session. The order of the two tasks was counterbalanced across batters. The batters stood in the batter's box and were asked to press a button held by the dominant hand as accurately and rapidly as possible whenever the pitcher threw a fastball, but not when the pitcher threw a slowball. Batters were asked to place equal weight on both accuracy and rapidity. Each batter viewed 10 fastballs and 10 slowballs thrown by the pitchers at random. The pitcher's camera (Sports Coaching Cam, JVCKENWOOD Corp.) was placed 8 m from the pitching rubber to the right of the pitcher to record the pitching motion at a sampling frequency of 240 Hz and detect the time when the pitcher released the ball. When the batter pressed the button, an LED installed in front of the pitcher's camera lit up. The time between the pitcher's ball release and LED lighting up were identified from video images.

#### Data Analysis

We employed the signal detection theory to evaluate the batters' judgment accuracy in the button pressing task (Stanislaw and Todorov, 1999). We calculated the hit rate (HR), false alarm rate (FAR), and criterion (c) in accordance with this theory. HR referred to the proportion of hits that responded correctly to a fastball. FAR referred to the proportion of false alarms, or incorrect responses to a slowball. The response bias was referred to as c, calculated as $c=-\frac{\left[z\left(HR\right)+z\left(FAR\right)\right]}{2}$. The z transformation referred to the conversion of HR or FAR to a z-score. A negative c value showed a response biased toward “True,” i.e., a fastball, and a positive c value shows one biased toward “False,” i.e., a slowball. The rapidity of judgement was quantified as reaction time (RT), which was calculated as the mean pressing time for a fastball aligned with the time the pitcher released the ball.

### Path Analysis

**Figure 2:**
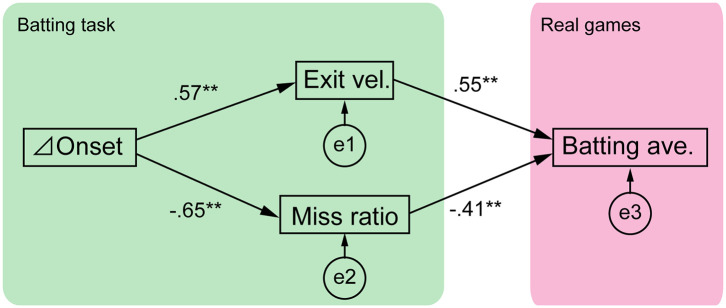
Structure of cognitive-motor processes in softball batting. The path diagram is shown with standardized path coefficients. e1-3 are the error terms. The model had good fit (χ2 test, *p* = 0.46; CFI = 1.00; TLI = 1.04; RMSEA = 0.00), and all the path coefficients were significant (**p < 0.01).

We used path analysis to clarify the structure of cognitive-motor processes in softball batting (lavaan R Ver. 3.4.2) (Rosseel, 2014). Path analysis is a statistical approach for investigating causal relationships among measured variables and it is also a type of SEM (Ullman and Bentler, 2004). Path diagrams are fundamental to SEM because they allow researchers to produce a diagram of a hypothesized set of relationships. The models of cognitive-motor structure using the path diagram are shown in Figures 2, 5, and these were established based on the hypotheses described in the Introduction section. The model in Figure 2 described the relationship between the delta onset and the batting outcomes of the batting task (exit velocity and miss ratio), and the relationship between the experimental outcomes and the batting average. Another model in **Figure 5** described the relationship between the cognitive scores (response accuracy and rapidity) of the button pressing task and the batting average. We evaluated our models using the maximum likelihood method. In SEM, a model is considered a good fit if the value of the chi-square test is insignificant. In addition, fit indices are often used for model evaluation. We evaluated our models using the chi-square test and three indices: the comparative fit index (CFI), the Tucker-Lewis index (TLI), and the root mean square error of approximation (RMSEA). CFI and TLI values exceeding 0.95 and an RMSEA value of 0.05 or less were considered to represent good fit. We also calculated the R-square values for all the endogenous variables, namely, the exit velocity, miss ratio, and batting average presented in Figure 2, and the batting average presented in **Figure 5**. That is, two types of values were calculated for the batting average: the experimental batting outcomes obtained from the batting task (Figure 2) and the response accuracy and rapidity obtained from the button pressing task (**Figure 5**).

## Results

### Influence of Cognitive-Motor Behaviors on the Batting Average

We investigated the structure of cognitive- motor processes using path analysis based on four explanatory variables: delta onset, exit velocity, miss ratio and batting average (Figure 2). The mean ± SD values of these variables were 25.1 ± 6.5% for the delta onset, 97.3 ± 6.6 km/h for exit velocity, 0.118 ± 0.169 for miss ratio, and 0.244 ± 0.112 for the batting average. The results of path analysis showed that the statistical tests and total fit scores indicated the model had good fit (χ^2^ test, *p* = 0.46; CFI = 1.00; TLI = 1.04; RMSEA = 0.00). All the path coefficients were significant (*p* < 0.01). The relationships between variables in the path analysis are shown as scatter plots in Supplementary Figure 1.

The TTCs between pitch speeds was 40%, therefore if a batter could shift the swing onset perfectly, the delta onset should also be 40%. As seen in Figure 1, batter E exhibited a larger delta onset while batter B exhibited a smaller one. We expected that the delta onset would influence the batting outcomes, which were evaluated as the exit velocity and miss ratio. Attaining a high exit velocity is one of a batter's primary goals because a ball hit with high exit velocity will travel a longer distance and penetrate the opponent's defenses. However, the exit velocity cannot be calculated if the batter performs a missed swing. Missed swings must be evaluated because these may be caused by the time gap between the two types of pitches. Therefore, we calculated both the missed swing ratio and exit velocity. As we expected, the delta onset was significantly related to both the exit velocity (*R*^2^ = 0.33) and miss ratio (*R*^2^ = 0.42) (Figure 2), suggesting that delta onset is a key element in the softball batting.

Furthermore, it was expected that the exit velocity and the miss ratio would correlate with the batting average in 2017 season. The result showed that both experimental outcomes were significantly correlated to the season batting average (*R*^2^ = 0.67) (Figure 2), suggesting that these experimental outcomes can partially predict a batter's performance in real games.

### Comparison of Batting Outcomes Between Groups With Large and Small Delta Onsets

**Figure 3:**
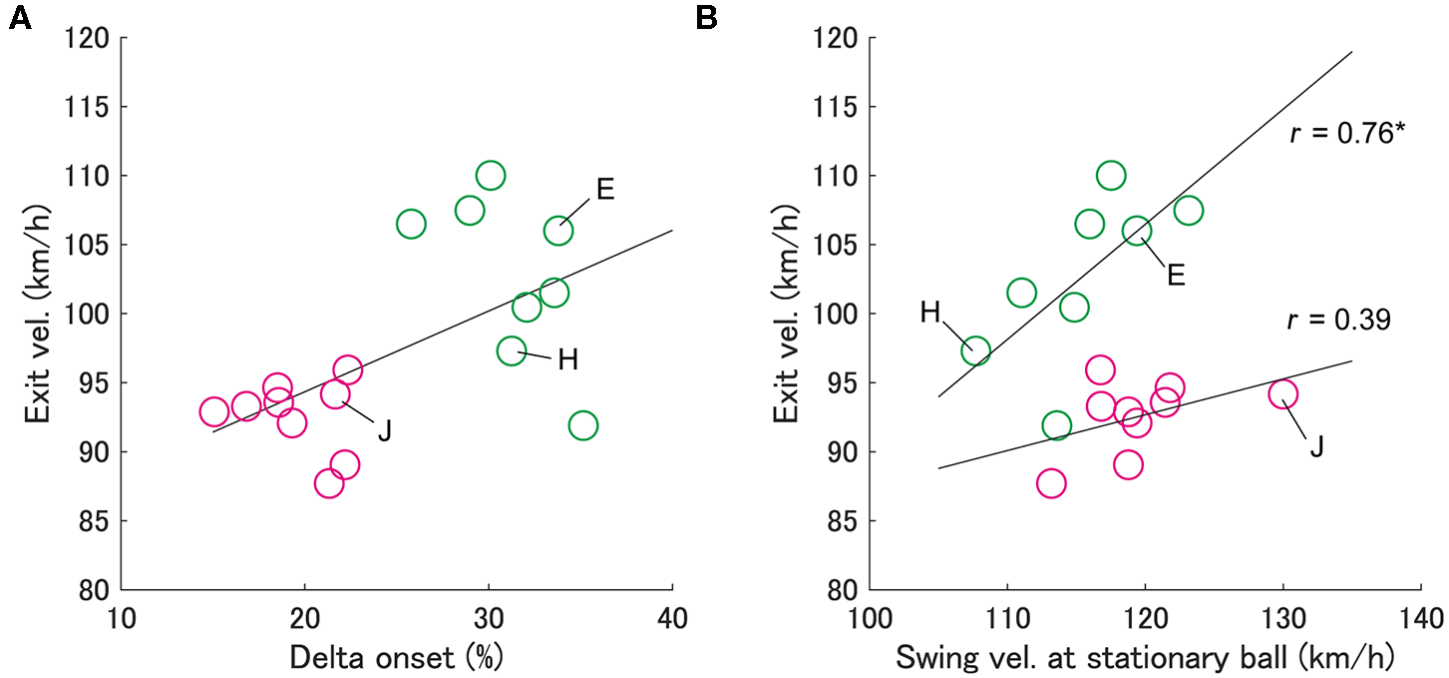
Relationship between exit velocity and delta onset **(A)** and swing velocity at stationary ball **(B)**. Each circle indicates an individual batter's data. Green circles indicate group L and magenta circles indicate group S, into which batters were divided based on the delta onset. In panel (b), it is shown that the exit velocity for group L is significantly correlated with the swing velocity at stationary ball (*p < 0.05), but not for group S.

**Figure 4:**
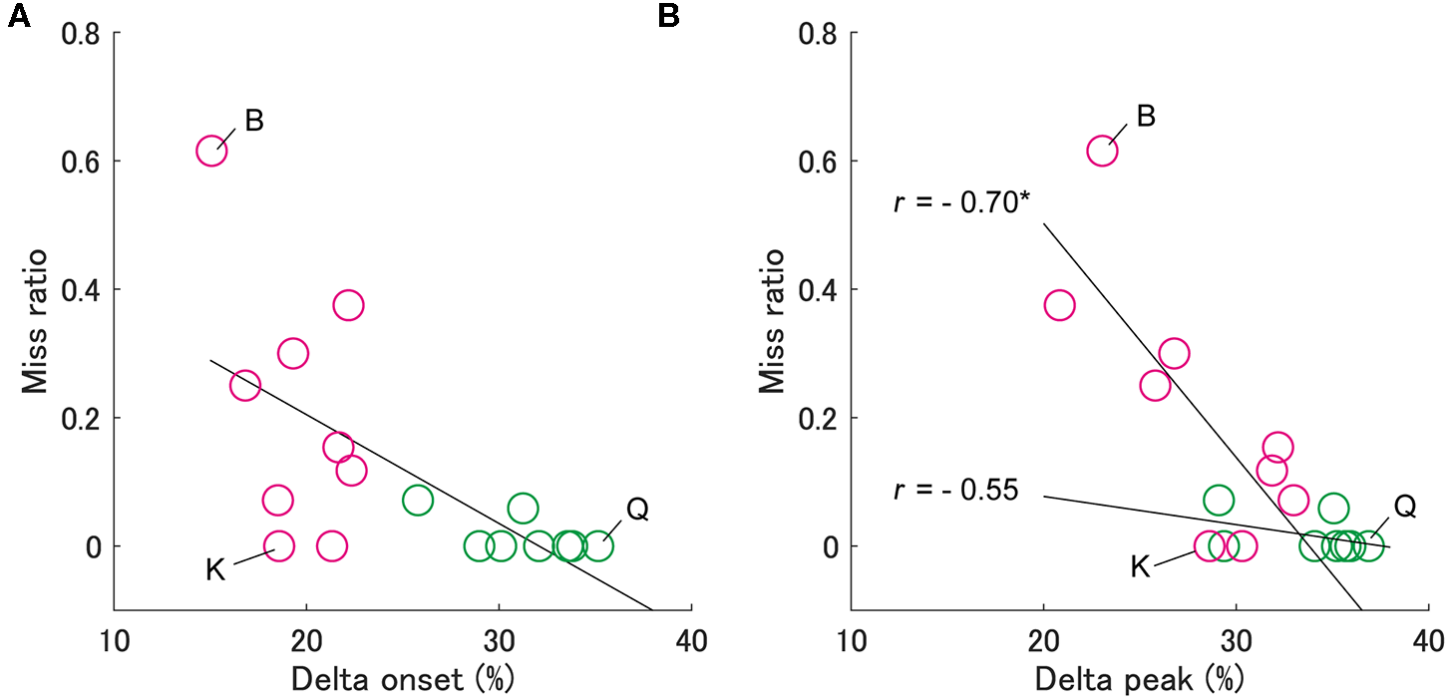
Relationship between the miss ratio and the delta onset **(A)** and the delta peak **(B)**. The notation is the same as in Figure 3. In panel (b), the miss ratio for group S is significantly correlated with the delta peak (*p < 0.05), but not for group L.

The contributions of the delta onset to the two batting outcomes (exit velocity and miss ratio) were significant but not large (*R*^2^ = 0.33 and 0.42, respectively) which could be due to interpersonal variability. We divided the batters into two groups based on delta onset (L: larger delta onset group 31.0 ±2.9 %, *N* = 8. S: smaller delta onset group 19.7 ±2.5 %, *N* = 9. two-sample *t*-test, *p* < 0.05) (magenta and green circles shown in Figures 3, 4). The comparison of variables between groups were shown in Supplementary Table 1. The interpersonal variability in terms of the exit velocity and miss ratio were different for each group (SD of the exit velocity: L = 6.0 km/h, S = 2.7 km/h. SD of the miss ratio: L = 0.03, S = 0.20). We then examined the possible causes of variability.

First, we examined the effect of the delta onset on exit velocity (Figure 3). Since it has been considered that the exit velocity is strongly influenced by the swing velocity (Sawicki et al., 2003), we examined the relationship between the exit velocity and the “swing velocity at stationary ball” for each group (Figure 3B). Since the swing velocity at stationary ball was measured when batters hit stationary balls in another session, it can be assumed that any differences were due to physical and technical factors rather than a cognitive factor. It was shown that the swing velocity at stationary ball correlated significantly with the exit velocity in group L (Spearman's r = 0.76, *p* < 0.05), but not significant in group S (Spearman's r = 0.38, *p* = 0.31). Batters with a larger delta onset (group L) and those with a larger swing velocity at stationary ball had a larger exit velocity (e.g., batter E in Figure 3). On the other hand, the batters with a small delta onset (group S) did not produce a large exit velocity even if they had a swing velocity at stationary ball similar to that of the batters in group L (e.g., batter J).

Second, we examined the effect of delta onset on the miss ratio (Figure 4). It is necessary to adjust the swing motion after the swing onset in an ongoing fashion to avoid executing a missed swing despite failing to identify the pitch type. To evaluate this ongoing adjustment, we calculated the delta peak as the difference between the peak time of hand velocities for fastballs and slowballs, similar to the delta onset. The delta peaks correlated significantly with the miss ratio in group S (Spearman's r = −0.70, *p* < 0.05) but not in group L (Spearman's r = −0.55, *p* = 0.18) (Figure 4B). This means that despite having smaller delta onsets, the batters from group S who had larger delta peaks produced smaller miss ratios (e.g., batter K in Figure 4). On the other hand, the batters in group L, who had a larger delta onset and delta peak, scarcely missed a swing (e.g., batter Q).

### Influence of Cognitive Scores on the Batting Average

In the button pressing task, participants were asked to press a button as accurately and rapidly as possible whenever the pitcher threw a fastball. The mean ± SD values of HR, FAR, c and RT were 0.90 ± 0.16, 0.38 ± 0.20, −0.71 ± 0.51, and 263 ± 58 ms, respectively. Compared to HR, FAR was more inaccurate overall, though HR was more accurate at higher values while FAR was more accurate at lower values. Moreover, c for 15 of 17 batters had negative values, i.e., their responses were biased toward the fastballs. These results indicate that the batters tended to anticipate a fastball. Thus, FAR was regarded as an index of judgment accuracy, while RT was regarded as an index of judgment rapidity. Furthermore, RT refers to judgment time, and not the onset time of swing motion, because the mean RT was earlier than the mean actual time of swing onset in the batting task (335.9 ± 29.4 ms for fastballs; 447.0 ± 35.2 ms for slowballs).

Judgment accuracy and quickness are often considered to be in a trade-off relationship. In the current study, however, FAR and RT were not significantly correlated (Pearson's *r* = −0.19, *p* = 0.46). None of the batters in our study seemed to use the potential strategy of delaying judgment to improve accuracy.

**Figure 5:**
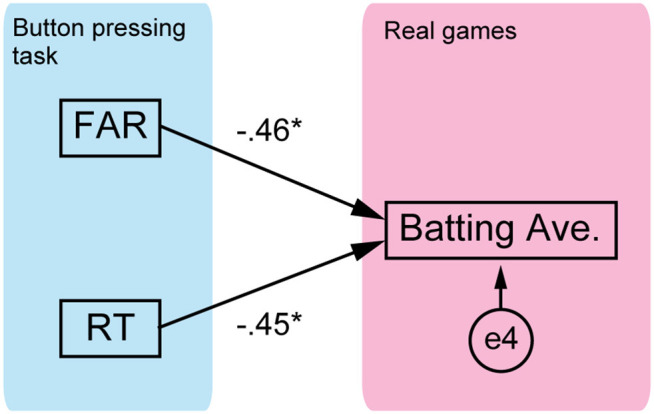
The direct relationship between the button pressing task and the batting average. The path diagram is shown with standardized path coefficients. e4 is the error term. Two path coefficients were both significant (*p < 0.05).

We investigated whether the judgement accuracy and rapidity explain the batting average (Figure 5). We found that both FAR and RT were significantly related to the batting average (*R*^2^ = 0.34), but these could only explain 34% of the batting average, which was half of what the outcomes in the batting task could explain (67%).

## Discussion

We investigated the structure of cognitive-motor processes in softball batting. We found that the batters who had a larger delta onset performed very well in the batting task (i.e., higher exit velocity and lower miss ratio). We also found that experimental outcomes in the batting task explained 67% of the batting average in real games, while the cognitive scores in the button pressing task explained only 34% of it. In addition, our batters could be divided into two groups based on delta onset and batters had various batting strategies.

### Delaying Swing Onset for Slowball Is the Key Element

The results of this study suggest that increasing the delta onset is one of the important factors in batting. There are two ways to increase the delta onset: the first is to make the swing onset earlier for fastball, and the other is to delay the swing onset for a slowball. In the current study, the results of the additional analysis showed that the swing onset time for slowballs in group L was significantly later than in group S (L: 106.0 ± 5.3%, S: 97.9 ± 3.1%, two sample *t*-test, *p* < 0.01), but that the onset time for fastballs was not different between groups (L: 74.6 ± 4.5%, S: 78.4 ± 3.3%, two sample *t*-test, *p* = 0.08). This means that batters who had a large delta onset delayed their swing onset for slowballs. In previous studies, batters were considered to expect a fastball when facing various ball speeds (Cañal-Bruland et al., 2015) because expecting slower balls would delay reaction time to unexpected fastballs. This finding was supported by our result that most batters were biased toward fastballs in the button pressing task. Moreover, many studies have reported that an expert's inhibitory process differs from that of a novice in psychophysics (Kida et al., 2005; Nakamoto and Mori, 2008, 2012; Nakamoto et al., 2013; Muraskin et al., 2015) and in simulated batting studies (Gray, 2009). Taken together, it can be said that superior batters' initial instincts are to respond to fastballs and they could also transiently inhibit their response to slowballs.

In order to delay the swing onset for slowball, batters should be able to anticipate the pitch type accurately and rapidly. A potential source of visual information for discrimination is the pitcher's motion and/or the initial ball trajectory. The participants' mean RT in the button pressing task was about 260 ms, which is similar to results from a previous study (about 240 ms for a fastball in Radlo et al., 2001). Another study reported that the Go/Nogo RT was about 290 ms when baseball players were asked to press a button when presented with a light stimulus (Kida et al., 2005). The fact that the RT in our study was less than that for a light stimulus suggests that there is information to predict the pitch type presented near or before the time that the pitcher releases the ball. Ranganathan and Carlton replaced the pitcher's motion and ball trajectory in virtual baseball batting and revealed that batters' stepping patterns are related to the pitcher's motion (Ranganathan and Carlton, 2007). More recently, Kimura et al. directly verified the effect of pitching motion on batting timing control by manipulating the combination of pitching motion and ball trajectory in a virtual reality environment (Kimura et al., 2018). Therefore, some batters in our study may use the pitcher's motion to anticipate the pitch type. Given the relationship between human visuomotor delay and TTC, this predictive ability may be an important factor in terms of shifting the swing onset.

### Various Batting Strategies

As presented in Figure 3, we qualitatively found three types of batters who were characterized by motion execution and physical abilities. First, batter E (Figure 1) discriminated the swing onset and had high swing velocity at stationary ball and exit velocity. Second, batter H discriminated the onset well but had the lowest swing velocity at stationary ball among all batters, while her exit velocity was intermediate. Third, batter J could not discriminate the onset well, and her exit velocity was low despite her swing velocity at stationary ball being the highest. Batters E and H performed a pre-programmed motor pattern that was not modulated by a cognitive function. Therefore, it was considered that the intrinsic biomechanical element (swing velocity at stationary ball) was reflected in the exit velocity. On the other hand, batter J did not show a high exit velocity because she was unable to perform the intrinsic motor pattern because of their inferior cognition. Therefore, the required trainings to heighten batting performance would be different depending on the characteristics of batters: Batter H might need strength and skill training to increase swing velocity, and batter J might need cognitive training.

Similarly, as presented in Figure 4, we found three types of batters who were characterized by motion execution and temporal adjustment abilities. First, batter Q could discriminate her swing onset and accurately performed a pre-programed motor pattern, therefore she responded appropriately to the temporal gap between two types of TTC and hardly missed a swing. Second, batter K could not discriminate her onset well, but she compensated for poor judgment by making an ongoing adjustment after the swing onset, therefore, she could avoid missing swings. Third, batter B (Figure 1) was incapable of either discrimination or ongoing adjustment, so she had more missed swings. Ijiri et al. also discovered two types of strategies (onset control vs. ongoing adjustment) to respond to different TTCs in a batting-simulated rapid interceptive task (Ijiri et al., 2014), although their study was conducted by employing only elbow flexion in a sitting position. It is important that results similar to those observed in a laboratory setting are obtained in an actual batting situation. Ijiri et al. also revealed that some participants who used the swing onset control strategy shifted to another strategy when time constraints became severe. The ability to perform ongoing adjustment will be important while predicting pitch trajectory is difficult for an individual.

### Prediction of Real Game Performance From Experimental Scores

Two batting outcomes obtained from the batting task could explain 67% of the batting average in real games. It is surprising that variables obtained from a simple task in a laboratory setting can explain so much about an individual's batting ability in real games, where extremely complex factors are involved. This result suggests that our experimental setup, a batting task including a cognitive aspect, is suitable for evaluating a batter's actual ability. Recently, it was reported that cognitive ability and batting performance in real games could be improved by incorporating a video occlusion task (Fadde, 2016; Müller et al., 2016). Those studies suggested that cognitive training might have a positive effect on batting performance, although detailed factors relating to cognition and batting performance were unknown. From these results, it was considered that cognitive ability is the basis of batting action and performance. However, in the current study, the button pressing task alone only predicted 34% of the batting average. This was because some batters adopted a strategy of compensation through ongoing adjustment, even though they were inferior in terms of cognition. The results demonstrated that more detailed individual characteristics can be grasped through evaluating the batting task including the cognitive aspects.

Our results will have an impact on cognitive and motor science research as well as on the actual sports scene. Research has already revealed amazing human cognitive and motor abilities, but there is skepticism toward the extent to which those results can be applied to complex actual situations such as in sports (Güldenpenning et al., 2017). Our experimental procedure and analysis method might fill this gap. We introduced path analysis using SEM and clarified the structure including the variables by performing a basic experiment, a sports experiment, and real games. Furthermore, we not only confirmed that many results of previous studies are partly meaningful in actual situations, but we also found results that can only be obtained from actual situations. Although the SEM is rarely applied in analyzing human cognitive-motor processes, it may be suitable for understanding complicated human systems. In addition, our findings have implications on actual sports scenarios. Grasping the cognitive and motor characteristics of individual players, which can explain performance in real games, would greatly help both players and coaches. We believe that our study will be serve as a catalyst for connecting basic research and sports scenarios.

## Data Availability Statement

The datasets generated for this study are available on request to the corresponding author.

## Ethics Statement

The studies involving human participants were reviewed and approved by Ethics and Safety Committees of NTT Communication Science Laboratories. The patients/participants provided their written informed consent to participate in this study.

## Author Contributions

DN, MY, and TK designed the experiments. DN, MY, AK, and TK performed the experiments. DN and AK performed the analyses. DN made the figures and wrote the paper. All authors contributed to discussion.

## Conflict of Interest

All authors are employees of NTT Communication Science Laboratories, which is a basic-science research section of Nippon Telegraph and Telecommunication corporation (NTT). This does not alter the authors' adherence to policies of Frontiers.
