# Supplementary material for: Behavioral Measures in a Cognitive-Motor Batting Task Explain Real Game Performance of Top Athletes
Source: Front Sports Act Living. 2020 May 12;2:55. doi: 10.3389/fspor.2020.00055 (PMC7739838; doi:10.3389/fspor.2020.00055)
Supplement: Supplementary file 1 [file Data_Sheet_1.docx]

Supplementary Material


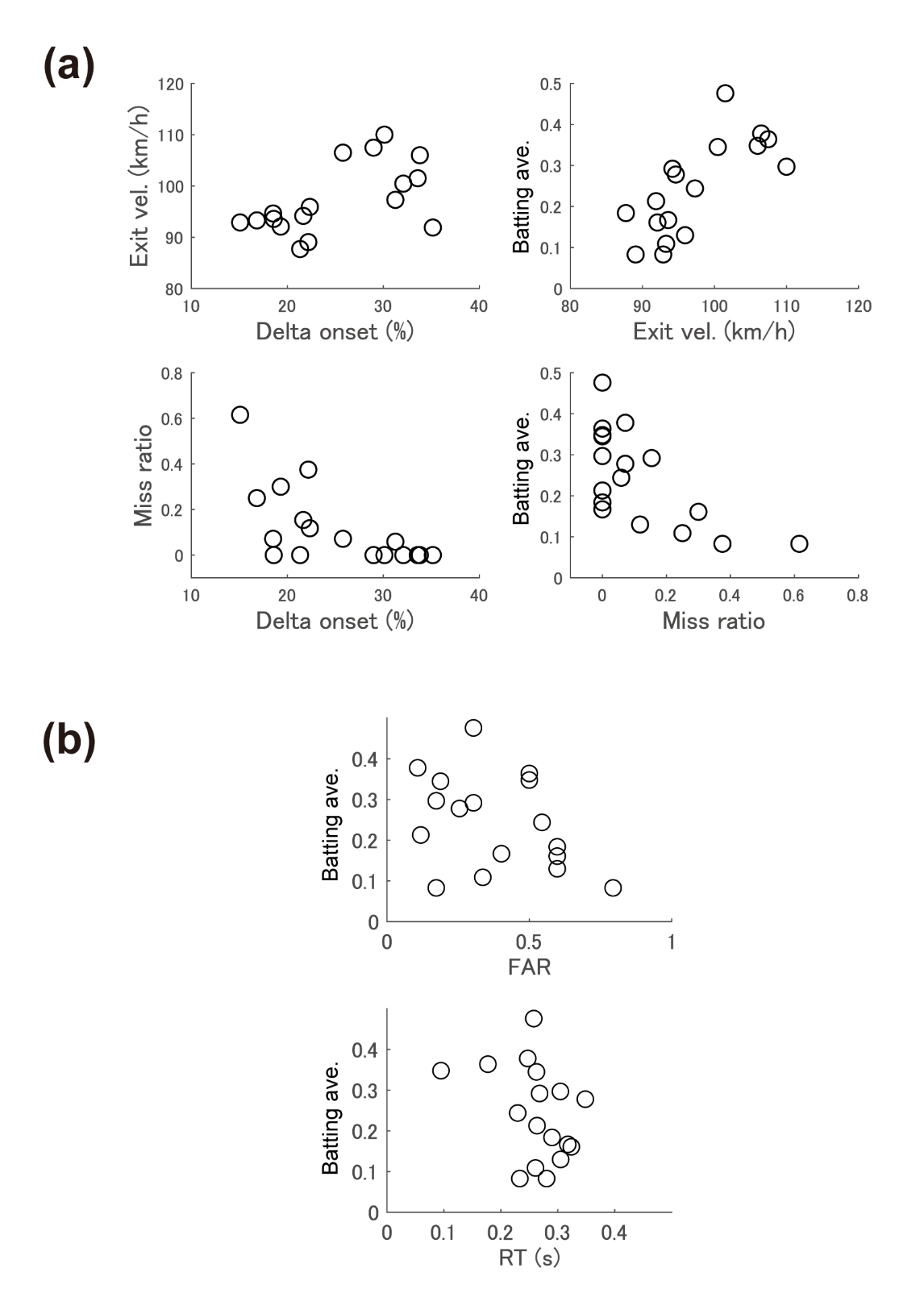


**Supplementary Figure 1.** Relationships between the variables in the path analyses. (a) The scatter plots between variables in cognitive-motor structure (Figure 2). (b) Scatter plots between variables in direct contribution of the button pressing task to the batting average (Figure 5).

**Supplementary Table 1.** Results for all variables (mean ± SD)

|  | All batters | group L | group S |  |
| --- | --- | --- | --- | --- |
| < Real games > |  |  |  |  |
| Batting ave. | 0.244 ± 0.112 | 0.333 ± 0.077 | 0.165 ± 0.072 | * |
| < Batting task > |  |  |  |  |
| Exit vel. [km/h] | 97.3 ± 6.6 | 102.6 ± 5.6 | 92.6 ± 2.5 | * |
| Miss ratio | 0.118 ± 0.169 | 0.016 ± 0.028 | 0.209 ± 0.189 | * |
| Swing vel. at stationary ball [km/h] | 117.7 ± 4.9 | 115.4 ± 4.5 | 119.7 ± 4.4 |  |
| Delta onset [%] | 25.1 ± 6.5 | 31.3 ± 2.8 | 19.5 ± 2.4 | * |
| Onset time for fastball | 76.6 ± 4.3 | 74.6 ± 4.5 | 78.4 ± 3.3 |  |
| Onset time for slowball | 101.7 ± 5.9 | 106.0 ± 5.3 | 97.9 ± 3.1 | * |
| Delta peak [%] | 30.8± 4.6 | 33.9 ± 2.8 | 28.1 ± 4.0 | * |
| Peak time for fastball | 93.7 ± 4.5 | 91.2 ± 2.8 | 95.9 ± 4.6 | * |
| Peak time for slowball | 124.5 ± 2.7 | 125.1 ± 1.7 | 123.9 ± 3.3 |  |
| < Button pressing task > |  |  |  |  |
| HR | 0.90 ± 0.16 | 0.89 ± 0.19 | 0.91 ± 0.13 |  |
| FAR | 0.38 ± 0.20 | 0.31 ± 0.17 | 0.45 ± 0.19 |  |
| Criteria | -0.71 ± 0.51 | -0.58 ± 0.56 | -0.83 ± 0.43 |  |
| RT [ms] | 263 ± 58 | 230 ± 61 | 292 ± 34 | * |

Note: Variables used in path analysis are indicated in bold font. Batters were divided into two groups (L: larger delta onset group, S: smaller delta onset group) for additional analysis in the Results section. *: significant difference between the two groups (p < 0.05).
